# Lingual bone thickness in the apical region of the horizontal mandibular third molar: A cross-sectional study in young Japanese

**DOI:** 10.1371/journal.pone.0263094

**Published:** 2022-01-25

**Authors:** Shinpei Matsuda, Hitoshi Yoshimura

**Affiliations:** Unit of Sensory and Locomotor Medicine, Division of Medicine, Department of Dentistry and Oral Surgery, Faculty of Medical Sciences, University of Fukui, Fukui, Japan; International Medical University, MALAYSIA

## Abstract

**Background:**

Perforation of the lingual plate in the apical region of mandibular third molars will increase the risk of aberration and migration of the root tip and the risk of lingual nerve injury. The aim of this study was to analyze anatomical information, including relationships between the apical region of horizontally impacted mandibular third molars and lingual plates, in young Japanese.

**Methods:**

Japanese patients, with horizontally impacted third molars, who underwent CT examination as a preoperative assessment for mandibular third molar extraction were included, and anatomical characteristics in the apical region of the right mandibular third molar were analyzed, in this study.

**Results:**

A total of 121 patients were included based on the inclusion and exclusion criteria of this study. The mean and standard deviation of the bone thickness on the lingual side of the mandibular third molar in the apical region was 1.5 ± 1.6 mm, and the absence of lingual cortical bone in the apical region, namely, “perforation”, was observed in 44 patients. The statistical analysis revealed the predictors of cases with perforation as follows: gender, age, and the available space evaluated by Pell and Gregory classification.

**Conclusions:**

This study clarified that “perforation” was sometimes observed in young Japanese, and that the predictors of those cases were as follows: gender, age, and the available space evaluated by Pell and Gregory classification.

## Introduction

Extraction of mandibular third molars is one of the most common oral and maxillofacial surgical procedures. Complications related to anatomical structures and surgical procedures include lingual and/or inferior alveolar nerve injury, mandibular fractures, aberration of teeth, and so on [1–4]. Since the angulation and position of mandibular third molars vary, preoperative assessment based on the results of imaging examinations and careful surgical procedures are important [1–4]. Perforation of the lingual plate in the apical region will increase the risk of aberration and migration of the root tip and the risk of lingual nerve injury [1]. The three-dimensional evaluation of the mandibular canal and the lingual plate is not possible with orthopantomography and is one of the factors that strongly suggests the importance of preoperative computed tomography (CT) examination. A recent study performed in China based on cone beam computed tomography reported that the root apex of mesially and horizontally impacted third molars is close to the mandibular lingual plate [5].This report provided valuable information for oral surgeons; however, the ages of the subjects and the degree of impacted depth of the third molar varied [5]. Further study is needed to take into account the root growth of mandibular third molars and the age at which many patients wish to have their teeth extracted. In addition, there may be differences between the races, and the authors considered that it should be taken into account [6]. Additionally, horizontally impacted mandibular third molars sometimes require complex extraction steps, including separation of the crown and root during extraction, and have the risk of aberration from the mandible and migration into the floor of the mouth. Therefore, the authors considered that the third molars in these impaction conditions should be studied.

In this study, conducted in Japanese patients, anatomical information, including relationships between the apical region of horizontally impacted mandibular third molars and the lingual plate, was analyzed taking age into account.

## Materials and methods

### Participants and inclusion/exclusion criteria

#### Inclusion criteria

1. Patients who underwent CT examination (slice width: 1 mm) as a preoperative assessment for mandibular third molar extraction at the Department of Dentistry and Oral Surgery of the University of Fukui Hospital from January 2016 to December 2020 participated in this study.
2. Japanese males and females between the ages of 17 and 26 participated, taking into account periodontitis, dental caries, and root formation of the mandibular second and/or third molars.
3. Only the mandibular third molars on the right side were analyzed, to eliminate multiple data extractions from the same patients.
4. Patients with horizontally impacted third molars, namely, those with no space between the mandibular second molar and the occlusal surface of the mandibular third molar, participated.

#### Exclusion criteria

1. Cases with the presence of caries observed by CT in mandibular second and/or third molars. These might be related to changes in the tooth axis.
2. Cases with bone destruction around the third molars observed by CT due to benign or malignant lesions. These might be related to changes in the tooth axis.

### Data extraction

#### General characteristics of patients

The author (S.M.) investigated gender and age.

#### Anatomical characteristics in the apical region of the right mandibular third molar

**Fig 1:**
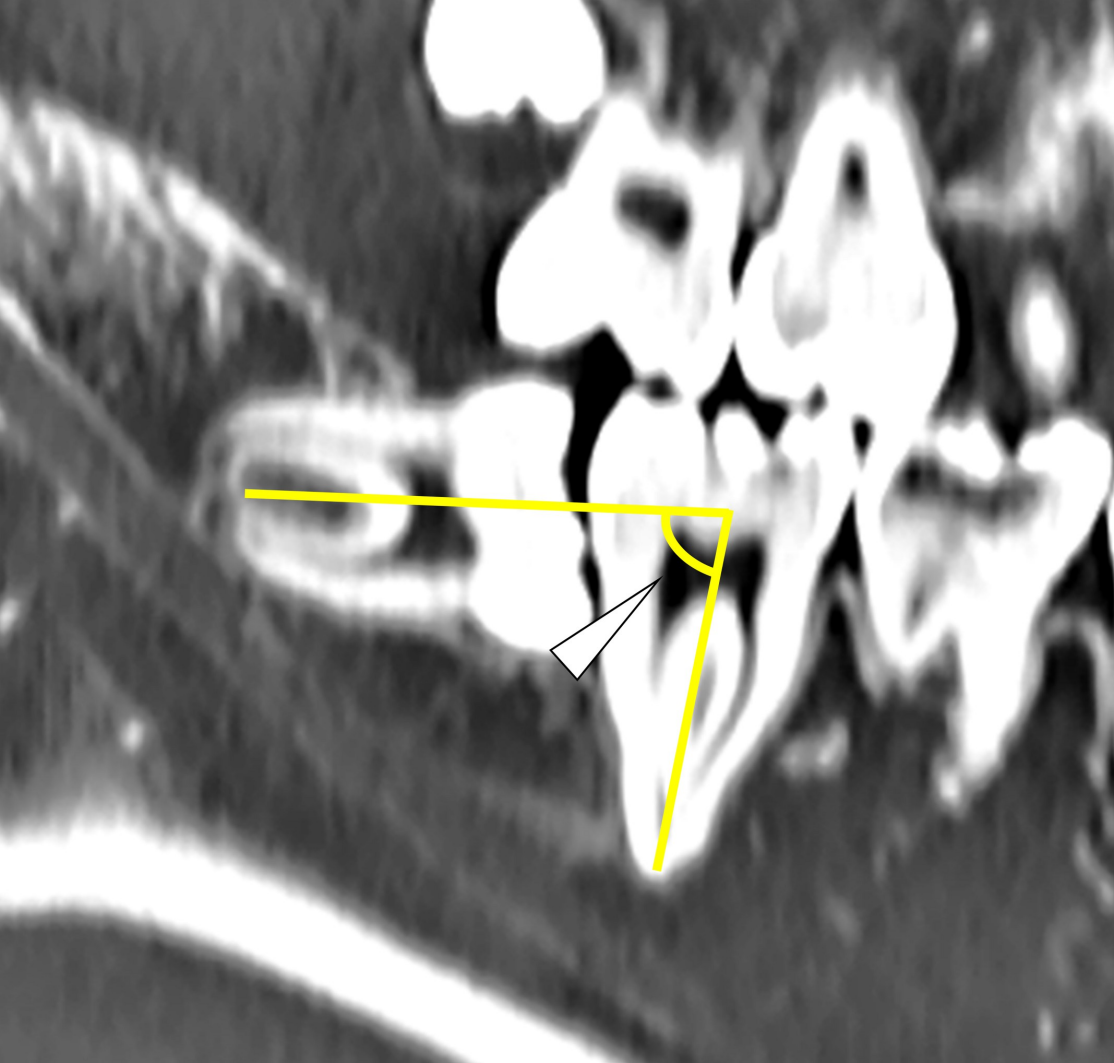
The angle formed by the tooth axis of the mandibular second molar and that of the mandibular third molar in a sagittal slice (the white arrow).

**Fig 2:**
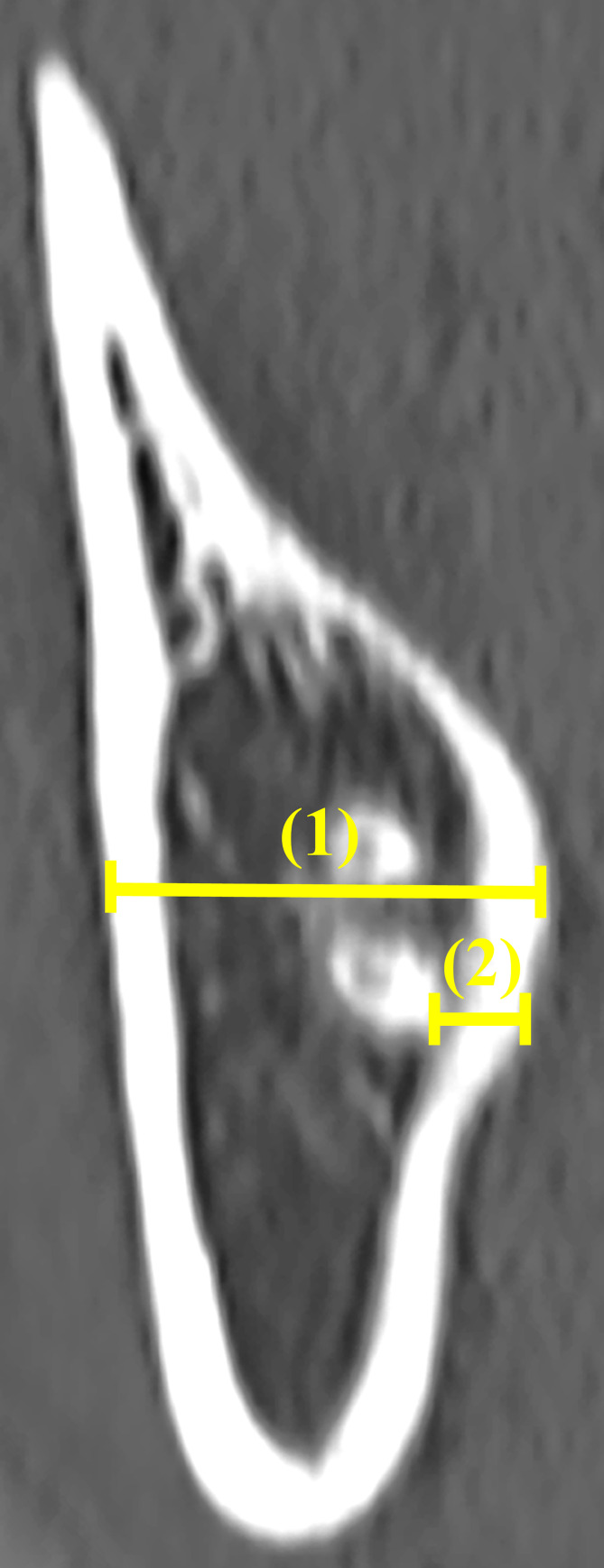
The bone thickness of the right mandible (1) and the bone thickness on the lingual side of the mandibular third molar (2) in the apical region in a coronal slice.

**Fig 3:**
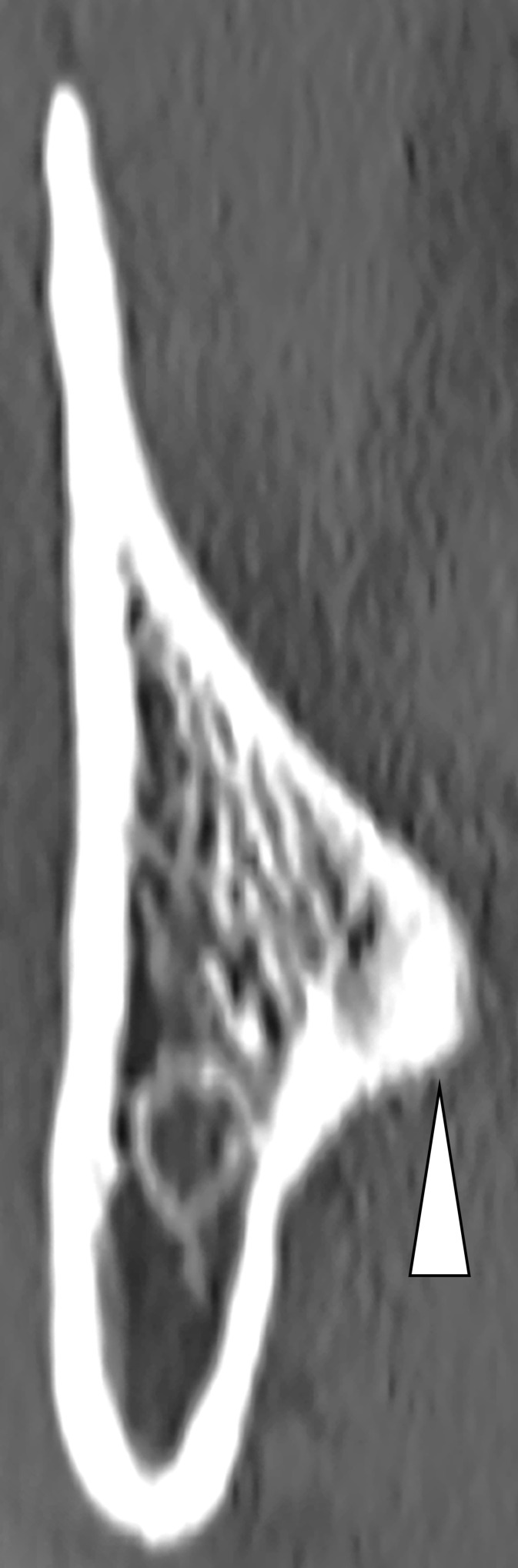
A case with “perforation” (with the white arrow indicating the root of mandibular third molar).

Based on CT imaging, the author (S.M.) investigated analytical information about the right horizontal third molar and mandible as follows:

1. The mandibular third molars were evaluated based on Pell and Gregory classification for impacted third molars as follows: Classes I, II, and III as available space and Levels A, B, and C as impaction depth [7].
2. The angle formed by the tooth axis of the mandibular second molar and that of the mandibular third molar in sagittal slices (Fig 1).
3. The length of the mandibular third molar, from the occlusal surface to the root apex, in sagittal slices.
4. Presence or absence of contact between the root of the mandibular third molar and the mandibular canal in coronal slices.
5. The bone thickness of the right mandible in the apical region in coronal slices (Fig 2).
6. Bone thickness on the lingual side of the mandibular third molar in the apical region in coronal slices. If lingual cortical bone was not observed in the apical region, it was defined as “perforation” in this study (Figs 2 and 3).

### Statistical analysis

Statistical analyses were performed using IBM SPSS Statistics 26 statistical software (IBM, Tokyo, Japan). *Mann-Whitney’s U-test* and the *chi-square test of independence* were used for statistical analysis, and *P < 0*.*05* was considered statistically significant.

### Ethics approval and informed consent

This study was a retrospective study approved by the Institutional Research Board (Ethical Committee of University of Fukui, Faculty of Medical Sciences; No. 20210006). There were no ethical issues in conducting this study because it was a retrospective study targeting examination images obtained for clinical treatment, and the authors declared that all methods were performed in accordance with the relevant guidelines and regulations (Declaration of Helsinki). Additionally, written informed consent was obtained from all participants at the time of dental examinations at the Department of Dentistry and Oral Surgery at the University of Fukui Hospital. For participants under the age of 18, informed consent was obtained from a parent and/or legal guardian. The Ethical Committee of the University of Fukui’s Faculty of Medical Sciences approved this procedure because the data for this retrospective study had been released to the public.

## Results

A total of 121 patients were included based on the inclusion and exclusion criteria of this study.

### General characteristics of the patients

The patients consisted of 53 males (43.8%) and 68 females (56.2%). The mean age and standard deviation of those patients were 22.3 ± 2.5 (17–26) years (S1 Table).

### Anatomical characteristics in the apical region of the right mandibular third molar

Regarding the available space and impaction depth based on Pell and Gregory classification, the available space was as follows: Class I: 70 (57.8%), Class II: 41 (33.9%), and Class III: 10 (8.3%) (S2 Table). Additionally, 69 patients were level A (57.0%), and 52 were level B (43.0%). Regarding the angle formed by the tooth axis of the mandibular second molar and that of the mandibular third molar, the mean value and standard deviation were 80.6 ± 8.0° (66.6–127.0°). The mean and standard deviation of the mandibular third molar length were 16.9 ± 1.3 mm (13.9–20.4 mm). The presence of contact between the root of the mandibular third molar and mandibular canal was observed in 54 patients (44.6%) (S3 Table). The mean and standard deviation of the bone thickness of the right mandible in the apical region were 13.5 ± 2.0 mm (9.4–18.7 mm). The mean and standard deviation of the bone thickness on the lingual side of the mandibular third molar in the apical region were 1.5 ± 1.6 mm (0–6.1 mm), and “perforation” was observed in 44 patients (36.4%).

The 44 patients with “perforation” consisted of 26 males (59.1%) and 18 females (40.9%). The mean age and standard deviation of patients with “perforation” were 23.1 ± 2.4 (18–26) years. Pell and Gregory classification, the mean value and standard deviation of the available space were as follows: Class I: 33 (75%), Class II: 9 (20.5%), and Class III: 2 (4.5%). Additionally, 30 patients were level A (68.2%), and 14 were level B (31.8%). The mean value and standard deviation of the angle formed by the tooth axis of the mandibular second molar and that of the mandibular third molar were 79.7 ± 5.6° (70.4–91°). The mean and standard deviation of the mandibular third molar length were 17.1 ± 1.2 mm (15.1–20.1 mm). The presence of contact between the root of the mandibular third molar and mandibular canal was observed in 15 patients (34.1%). The mean and standard deviation of the bone thickness of the right mandible in the apical region were 13.3 ± 1.9 mm (9.4–17.9 mm).

### Statistical analysis (between with “perforation” and without “perforation”)

**Fig 4:**
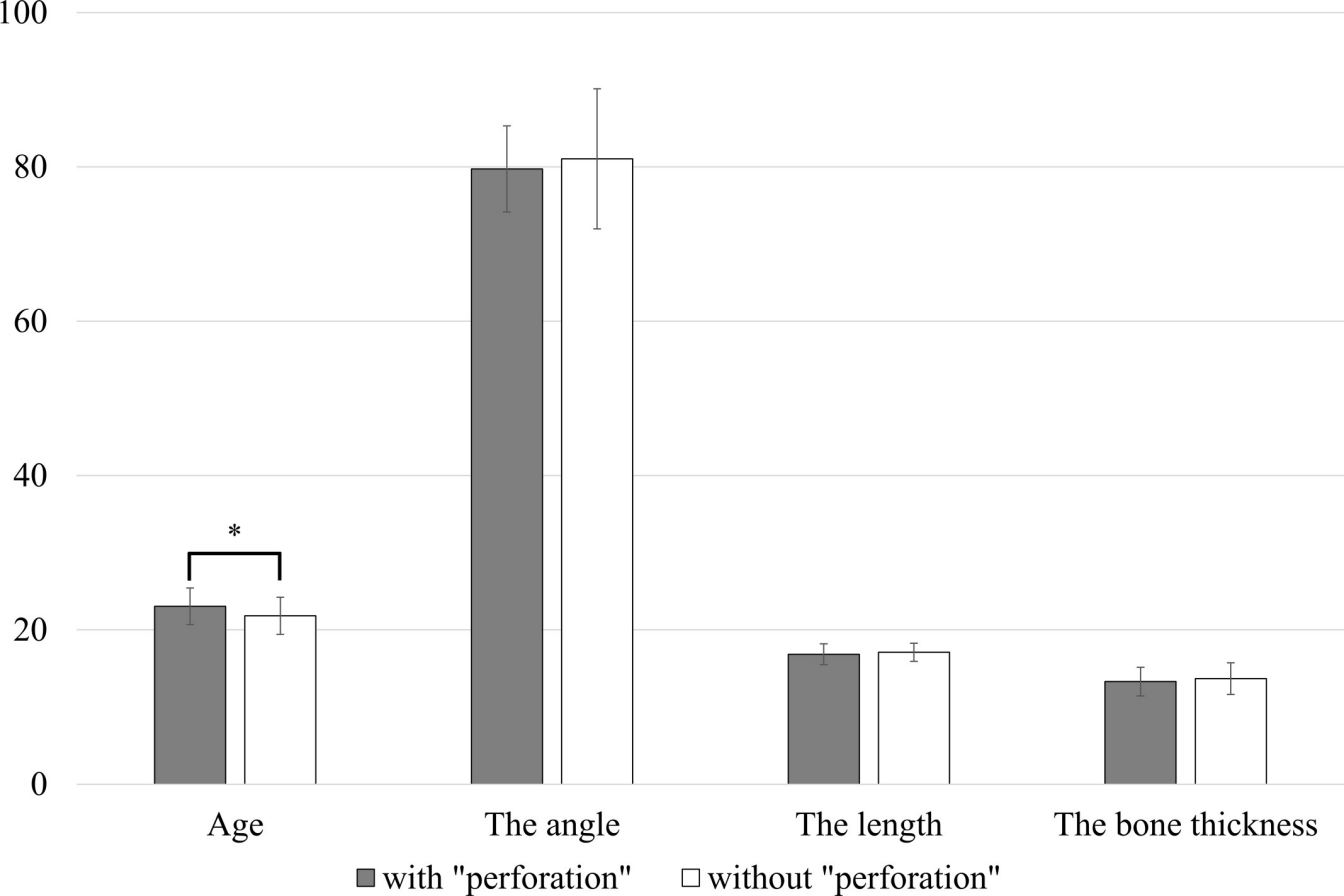
The comparison between patients with “perforation” and without “perforation” (*P < 0.05, *Mann-Whitney’s U-test*).

There was a significant relationship between the sex of patients without “perforation” (77 patients (27 males and 50 females)) and that of patients with “perforation” (*P* < 0.05, *chi-square test of independence*) (S4 Table). There was a significant difference between the age of patients without “perforation” (mean and standard deviation: 21.8 ± 2.4 years) and that of patients with “perforation” (*P* < 0.05, *Mann-Whitney U-test*) (Fig 4). There was a significant relationship between the available space of patients without “perforation” (Class I: 37 (48.0%), Class II: 32 (41.6%), and Class III: 8 (10.4%)) and that of patients with “perforation” (*P* < 0.05, *chi-square test of independence*). There was no significant relationship between the impaction depth of patients without “perforation” (Level A: 39 (50.6%) and Level B: 38 (49.4%)) and that of patients with “perforation” (*P* = 0.06, *chi-square test of independence*). There was no significant difference between the angle formed by the tooth axis of the mandibular second molar and that of the mandibular third molar of patients without “perforation” (Mean and standard deviation: 81.0 ± 9.1°) and that of patients with “perforation” (*P* = 0.46, *Mann-Whitney’s U-test*). There was no significant difference between the mandibular third molar length of patients without “perforation” (mean and standard deviation: 16.8 ± 1.3 mm) and that of patients with “perforation” (*P* = 0.49, *Mann-Whitney’s U*-test). There was no significant difference between the bone thickness of the right mandible in the apical region of patients without “perforation” (mean and standard deviation: 13.7 ± 2.1 mm) and that of patients with “perforation” (*P* = 0.32, *Mann-Whitney’s U-test*).

## Discussion

This study clarified that young patients sometimes lack lingual cortical bone in the apical region, namely, “perforation”. Based on this study, the authors thought that cases with “perforation” are not rare, although not as common as cases with the presence of contact between the root of the mandibular third molar and the mandibular canal. Therefore, CT is an important preoperative imaging examination for mandibular third molar extraction because “perforation” can be related to migration into the lingual anatomical organ and damage to the lingual nerve, and “perforation” cannot be observed by orthopantomography. Additionally, the authors recommended that even in cases in which extraction procedures of the mandibular third molar will be expected to be difficult based on preoperative imaging examinations, separation between the crown and root and separation between the mesial root and distal root should be avoided whenever possible to prevent root migration associated with “perforation”.

The statistical analysis of this study revealed the predictors of cases with perforation as follows: there was a significant relationship between gender, there was a significant difference between age, and there was a significant relationship between the available space of patients without “perforation” and that of patients with “perforation”. To the authors’ surprise, there was no significant relationship between impaction depth for patients without “perforation” and that of patients with “perforation”. Also surprising was the lack of significant difference between the two groups of patients with regard to the angle formed by the tooth axis of the mandibular second molar and that of the mandibular third molar, the mandibular third molar length, and the bone thickness of the right mandible in apical region.

Mandibular third molars are sometimes associated with the following conditions: dental caries, pericoronitis, root resorption, periodontitis, infections, cysts, tumors, and mandibular fractures [8].Therefore, without adequate dental maintenance, it is difficult to protect the mandibular third molar fully from those conditions [9]. Tai et al. reported that dental lesions were observed in approximately 7% and periodontal lesions were observed in approximately 40% of cases of impacted third molars; furthermore, dental and periodontal lesions in adjacent mandibular second molars were sometimes observed [10]. Additionally, they concluded that early prophylactic extraction of the mandibular third molar with mesioangular impaction could prevent impacted mandibular third molar-associated lesions in mandibular third and second molars [10]. With regard to impacted mandibular third molars, the necessity of extraction and the age at which the procedure should be performed are controversial. If patients choose to extract the impacted horizontal mandibular third molar at a relatively young stage, it is important to perform safe extractions after risk assessment based on appropriate preoperative evaluation by imaging examinations, including CT examination.

Extraction of mandibular third molars is a common oral and maxillofacial surgical procedure, as described above. Therefore, research and advances in extraction techniques and preoperative assessment should continue [11–15]. Recently, simulation of tooth extraction surgery based on three-dimensional image construction technology has also been reported, and innovation in tooth extraction surgery may occur with the development of digital technology [14, 15]. Yoo et al. reported on the application of artificial intelligence to predict the difficulty of surgery for third molar extraction [16]. The authors reported on an artificial intelligence-based analysis method for orthopantomography [17]. Artificial intelligence is increasingly being applied to the dental field [18]. In the relatively near future, artificial intelligence-based analysis of CT imaging obtained for mandibular third molar assessment will be a remarkable development. Advances based on these studies will contribute to reducing the physical burden on patients and preventing postoperative complications.

In this study, only Japanese patients with horizontally impacted third molars, namely, those with no space between the mandibular second molar and the occlusal surface of the mandibular third molar, participated. This study provided clinically novel and important information. On the other hand, racial differences in tooth length and mandibular bone morphology have been noted, and these must be declared limitations of this study [6]. Furthermore, the limitations of the methodology of this study were to perform sagittal and coronal slice CT examinations with a 1 mm slice width. Inclusion criteria were set by the authors as follows: patients between the ages of 17 and 26 participated, taking into account periodontitis, dental caries, and root formation of the mandibular second and/or third molars; however, there may be room for debate on this point as well. The limitation of this study is that there were no patients with Level C impaction depth. Further study of patients with impaction depth at Level C will be needed.

## Conclusion

This study clarified that “perforation” was sometimes observed in young Japanese, and that the predictors of cases with “perforation” were as follows: gender, age, and the available space evaluated by Pell and Gregory classification.

## Supporting information

S1 Table The general characteristics of patients. (DOCX)

S2 Table Anatomical characteristics of the right mandibular third molar. (DOCX)

S3 Table Anatomical characteristics in the apical region of the right mandibular third molar. (DOCX)

S4 Table The comparison with patients with and without “perforation”. (DOCX)

S1 File (XLSX)

## Abbreviations

CT: computed tomography
