# Correction: Lingual bone thickness in the apical region of the horizontal mandibular third molar: A cross-sectional study in young Japanese

**DOI:** 10.1371/journal.pone.0270541

**Published:** 2022-06-22

**Authors:** Shinpei Matsuda, Hitoshi Yoshimura

## Notice of republication

In light of an issue identified post-publication, this article was republished on February 7, 2022. Please download this article again to view the correct version.
