# Supplementary material for: Lingual bone thickness in the apical region of the horizontal mandibular third molar: A cross-sectional study in young Japanese
Source: PLoS One. 2022 Jan 25;17(1):e0263094. doi: 10.1371/journal.pone.0263094 (PMC8789189; doi:10.1371/journal.pone.0263094)
Supplement: S1 Table — (DOCX) [file pone.0263094.s001.docx]

**Table 1. The general characteristics of patients**

|  |  | **Cases (n)** | **(%)** |
| --- | --- | --- | --- |
| **Gender** | Male | 53 | 43.8 |
|  | Female | 68 | 56.2 |
|  | Total | 121 | 100 |
| **Age** |  |  |  |
|  | 17-21 | 46 | 38.0 |
|  | 22-26 | 75 | 62.0 |
|  | Total | 121 | 100 |
