# Supplementary material for: Lingual bone thickness in the apical region of the horizontal mandibular third molar: A cross-sectional study in young Japanese
Source: PLoS One. 2022 Jan 25;17(1):e0263094. doi: 10.1371/journal.pone.0263094 (PMC8789189; doi:10.1371/journal.pone.0263094)
Supplement: S2 Table — (DOCX) [file pone.0263094.s002.docx]

**Table 2. Anatomical characteristics of the right mandibular third molar**

|  | **Cases (n)** | **(%)** |
| --- | --- | --- |
| The available space on Pell and Gregory classification | | |
| Class I | 70 | 57.8 |
| Class II | 41 | 33.9 |
| Class III | 10 | 8.3 |
| The impaction depth based on Pell and Gregory classification | | |
| Level A | 69 | 57.0 |
| Level B | 52 | 43.0 |
| Level C | 0 | 0.0 |
| The angle formed by the tooth axis of the mandibular second and third molar | | |
| ≧70 | 5 | 4.1 |
| 70.1-80 | 56 | 46.3 |
| 80.1-90 | 50 | 41.3 |
| 90.1-100 | 9 | 7.4 |
| 100≦ | 1 | 0.8 |
| The length of mandibular third molar | |  |
| ≧14 | 2 | 1.7 |
| 14.1-16 | 29 | 24.0 |
| 16.1-18 | 68 | 56.2 |
| 18.1-20 | 20 | 16.5 |
| 20< | 2 | 1.7 |
