# Supplementary material for: Lingual bone thickness in the apical region of the horizontal mandibular third molar: A cross-sectional study in young Japanese
Source: PLoS One. 2022 Jan 25;17(1):e0263094. doi: 10.1371/journal.pone.0263094 (PMC8789189; doi:10.1371/journal.pone.0263094)
Supplement: S3 Table — (DOCX) [file pone.0263094.s003.docx]

**Table 3. Anatomical characteristics in the apical region of the right mandibular third molar**

|  | **Cases (n)** | **(%)** |
| --- | --- | --- |
| The contact between the root of mandibular third molar and mandibular canal | | |
| Presence | 54 | 44.6 |
| Absence | 67 | 55.4 |
| The bone thickness of the right mandible in apical region | | |
| ≧10 | 3 | 2.5 |
| 10.1-12 | 27 | 22.3 |
| 12.1-14 | 43 | 35.5 |
| 14.1-16 | 33 | 27.3 |
| 16.1-18 | 12 | 9.9 |
| 18.1≦ | 3 | 2.5 |
| The bone thickness on the lingual side of the mandibular third molar in apical region | | |
| 0 | 44 | 36.4 |
| 0.1-2 | 41 | 33.9 |
| 2.1-4 | 24 | 19.8 |
| 4.1-6 | 11 | 9.1 |
| 6.1≦ | 1 | 0.8 |
