# Supplementary material for: Lingual bone thickness in the apical region of the horizontal mandibular third molar: A cross-sectional study in young Japanese
Source: PLoS One. 2022 Jan 25;17(1):e0263094. doi: 10.1371/journal.pone.0263094 (PMC8789189; doi:10.1371/journal.pone.0263094)
Supplement: S4 Table — (DOCX) [file pone.0263094.s004.docx]

**Table 4. The comparison with patients with and without "perforation"**

|  |  | with "perforation | | without "perforation | |  |
| --- | --- | --- | --- | --- | --- | --- |
|  |  | **Cases (n)** | **(%)** | **Cases (n)** | **(%)** | ****P*** |
| Total |  | 44 | 36.4 | 77 | 63.6 |  |
| Gender | Male | 26 | 43.8 | 27 | 35.1 | *P<0.05* |
|  | Female | 18 | 56.2 | 50 | 64.9 |  |
| The available space on Pell and Gregory classification | | | | |  |  |
|  | Class I | 33 | 75 | 37 | 48.0 | *P<0.05* |
|  | Class II | 9 | 20.5 | 32 | 41.6 |  |
|  | Class III | 2 | 4.5 | 8 | 10.4 |  |
| The impaction depth based on Pell and Gregory classification | | | | | |  |
|  | Level A | 30 | 68.2 | 39 | 50.6 | *P=0.06* |
|  | Level B | 14 | 31.8 | 38 | 49.4 |  |
|  | Level C | 0 | 0 | 0 | 0 |  |

**Chi-square test of independence*
